# Iodine mouthwashes as deterrents against severe acute respiratory syndrome coronavirus 2 (SARS-CoV-2)

**DOI:** 10.1017/ice.2020.1356

**Published:** 2020-12-07

**Authors:** Claudio Blasi

**Affiliations:** Ospedale Sandro Pertini, Rome, Italy

*To the Editor—*Severe acute respiratory syndrome coronavirus 2 (SARS-CoV-2) enters the body via the eyes, nose, and mouth and infects the upper respiratory tract causing the initial clinical symptoms. After exposure, a period of incubation, mainly at the level of the oropharynx, lasts 5–6 days, allowing the viral replication to reach a number at which spread to the rest of the body occurs, giving rise to symptomatic coronavirus disease 2019 (COVID-19), organ damage, and/or death.

This incubation phase and progress of disease are dependent on the rate of viral replication; thus, a possible means to curb the proliferation of the virus after its entry into the upper respiratory tract is rinsing and gargling with iodine mouthwash. A number of in vitro studies support this possibility.

Enveloped viruses, like SARS-CoV-2, have an outer lipidic bilayer membrane that is very sensitive to antiseptic agents. Iodine is one of these agents, and its action is rapid, taking place through the degeneration of the nucleoproteins of viral particles.^1^ In influenza A viruses, this antiviral property occurs by inhibiting the activity of 2 glycoproteins on the viral surface: viral hemagglutinin binding activity and viral neuraminidase catalytic hydrolysis. These processes mediate, respectively, the entry of the virus into the host cells and its release and diffusion into other cells.^2^

With SARS-CoV-2, iodine could, in the same way, inhibit the activity of the surface glycoprotein that mediates its entry into the airway epithelium cells by connecting to the human angiotensin-2 converting enzyme (ACE2) receptor. Aqueous solutions of molecular iodine (I_2_) are unstable due to the volatility of iodine, and they can cause burning and cytotoxicity. For this reason, compounds called iodophores have been developed in which iodine is combined with neutral carrier polymers acting as solubilizing agents and as iodine reservoirs that keep the release of iodine low.

The most utilized iodine preparation is povidone-iodine (PVP-I) in which molecular iodine I_2_, in the form of I_3_^−^, is physically intercalated in the PVP helix via hydrogen bonds and is in chemical equilibrium with free I_2_ in solution; bound iodine is released from the PVP helix to maintain equilibrium as long as free I is utilized.^1^ In the aqueous medium, a chemical equilibrium develops in which only a ~1/1,000 part of the iodine is released and made available as free molecular iodine, which is responsible for germicidal activity.

PVP-I–based antiseptics have demonstrated a broad virucidal spectrum in vitro, both for enveloped and nonenveloped viruses.^3^

Following the H1N1 swine flu outbreak in 2009, the Japanese Ministry of Health recommended daily gargling with antimicrobial agents as a protective hygienic measure to prevent upper respiratory tract infection (URTI). This practice was supported by the results of studies that examined the role of gargling in healthy study participants and those with frequent or persistent respiratory tract infections.^4^

Numerous in vitro studies have shown a rapid virucidal activity of PVP-I contained in mouthwash solution against all respiratory pathogens tested according to the European standard requirements. A 7% concentration with 1:30 dilution has been recommended in Japan for common use (equivalent to a concentration of 0.23% of the active substance); a contact time of 15 seconds, which reflects a similar or even shorter duration time of gargling in real-life conditions, has proven sufficient.^5^ The PVP-I solution has demonstrated rapid antimicrobial activity similar to that shown in previous in vitro studies.^5^

This solution has been successfully tested against SARS-CoV-1 and Middle East respiratory syndrome (MERSCoV); it resulted in a 99.99% viral titer reduction.^5^ The same efficacy has also been shown in other in vitro studies with numerous products for pharyngal hygiene based on PVP-I at various concentrations (from 0.23% to 1%).^6^

This prompt antiviral activity has been confirmed in influenza enveloped viruses grown in embryo hen eggs and treated with 6 products containing PVP-I at a concentration of 0.23%. After only 10 seconds, the viral titers were reduced to levels below detection.^7^

Recently nasal and oral antiseptic formulations of PVP-I have been successfully tested in SARS-CoV2 with rapid, as short as 60 seconds, inactivation capacity of at concentrations up to 2.5%.^8^

The safety profile of PVP-I applied to the nasal and oral cavity has been confirmed after >60 years of use and is addressed by a vast literature.^8^ Unlike other oral antiseptic agents, it does not cause irritation or damage to the oral mucosa, even after prolonged use. Although iodine absorption may occur in the long term with the use of PVP-I, thyroid gland dysfunction is rare. Moreover, despite its long-term use, the development of resistance to PVP-I in microorganisms has not been reported.^2^ Finally, the common iodine–alcohol solutions diluted in water might also be effective but, due to their instability, they should be applied swiftly.

The in vitro efficacy demonstrated in various reliable studies suggests that PVP-I in oral solution could be an effective agent for the prevention of SARS-CoV-2 infections, reducing viral spread in situations of high risk of exposure to pathogens by oral and respiratory route.^2,9^ Together with hand washing and mask wearing, they could constitute an effective personal hygiene measure against airborne and droplet transmission by reducing the viral load in the oral cavity and in the oropharynx.

In view of the current absence of medication known to be effective at preventing and treating this highly contagious disease that has challenged traditional healthcare systems,^10^ iodophore mouthwashes might be candidates for fast-track approval as simple and inexpensive therapeutic compounds.
